# Characterization of the caprolactam degradation pathway in *Pseudomonas jessenii* using mass spectrometry-based proteomics

**DOI:** 10.1007/s00253-018-9073-7

**Published:** 2018-05-31

**Authors:** Marleen Otzen, Cyntia Palacio, Dick B. Janssen

**Affiliations:** Biochemical Laboratory, Groningen Biomolecular Sciences and Biotechnology Institute, University of Groningen, Nijenborgh 4, 9747 AG Groningen, The Netherlands

**Keywords:** Caprolactam, Caprolactamase, Omega aminotransferase, Mass spectrometry, Proteomics, *Pseudomonas jessenii*

## Abstract

Some bacterial cultures are capable of growth on caprolactam as sole carbon and nitrogen source, but the enzymes of the catabolic pathway have not been described. We isolated a caprolactam-degrading strain of *Pseudomonas jessenii* from soil and identified proteins and genes putatively involved in caprolactam metabolism using quantitative mass spectrometry-based proteomics. This led to the discovery of a caprolactamase and an aminotransferase that are involved in the initial steps of caprolactam conversion. Additionally, various proteins were identified that likely are involved in later steps of the pathway. The caprolactamase consists of two subunits and demonstrated high sequence identity to the 5-oxoprolinases. *Escherichia coli* cells expressing this caprolactamase did not convert 5-oxoproline but were able to hydrolyze caprolactam to form 6-aminocaproic acid in an ATP-dependent manner. Characterization of the aminotransferase revealed that the enzyme deaminates 6-aminocaproic acid to produce 6-oxohexanoate with pyruvate as amino acceptor. The amino acid sequence of the aminotransferase showed high similarity to subgroup II ω-aminotransferases of the PLP-fold type I proteins. Finally, analyses of the genome sequence revealed the presence of a caprolactam catabolism gene cluster comprising a set of genes involved in the conversion of caprolactam to adipate.

**Electronic supplementary material:**

The online version of this article (10.1007/s00253-018-9073-7) contains supplementary material, which is available to authorized users.

## Introduction

Caprolactam is a large-volume industrial compound mainly used in the production of Nylon 6, a versatile synthetic polymer applied in fabrics, utensils, mechanical parts, etc. Synthesis of Nylon 6 is achieved by ring-opening polymerization of caprolactam at high temperatures. During this process, several side products are generated, including 6-aminocaproic acid (6-aminohexanoic acid (6-ACA)) unreacted monomers, as well as dimers, cyclic dimers, and oligomers of 6-ACA. Caprolactam and these by-products are present as contaminants in waste water of nylon production plants (Fortmann and Rosenberg 1984; Kalinová et al. 2014, 2016). The toxic nature of these chemicals, including mutagenic effects and plant growth inhibition, has been shown in different studies (Sheldon 1989; Vogel 1989).

Several microorganisms are able to degrade caprolactam and/or these by-products, including strains of *Pseudomonas*, *Alcaligenes*, and *Acinetobacter* (Kulkarni and Kanekar 1998; Rajoo et al. 2013; Baxi and Shah 2002). Previously, a caprolactam degradation pathway was proposed (Caspi et al. 2014). It starts with a caprolactam-ring cleavage to form 6-ACA, followed by the deamination to 6-oxohexanoate and subsequent oxidation to yield adipate. Adipate can be further converted via β-oxidation reactions of the fatty acid metabolism pathway. Whereas this pathway seems straightforward, information about the proteins involved in bacterial caprolactam degradation is rare. At first sight, one would expect a hydrolytic enzyme involved in lactam ring opening. The conversion of the related d,l-α-amino-ε-caprolactam by a combination of a racemase and a l-amino acid lactam hydrolase to yield l-lysine has been described (Payoungkiattikun et al. 2017; Fukumura et al. 1978; Ahmed et al. 1982, 1986). Additionally, a putative hydrolase and aminotransferase have been reported for caprolactam metabolism. Recent work suggests that a dehydrogenase may oxidize 6-oxohexanoate to adipate in *Arthrobacter* sp. KI72 and in *Acinetobacter* sp. NCIMB 9871. Subsequently, an aminotransferase might catalyze the conversion of 6-ACA to 6-oxohexanoate in the cell (Takehara et al. 2018; Iwaki et al. 1999).

The environmental relevance of caprolactam, and the importance to understand the biodegradation pathway of this synthetic compound, prompted us to investigate the biochemistry of the early degradation steps. In this paper, we describe a newly isolated caprolactam degrading strain of *Pseudomonas jessenii*. To investigate the pathway, and identify the enzymes involved, we used label-free quantitative mass spectrometry-based proteomics (van der van der Wal and Demmers 2015; Fabre et al. 2014; Wasinger et al. 2013). From the various caprolactam-induced proteins, we further examined an unexpected ATP-dependent caprolactamase that forms 6-ACA and a class-II omega-aminotransferase that converts 6-ACA to 6-oxohexanoate. The activities of the enzymes were examined with heterologously expressed proteins. Furthermore, a caprolactam degradation gene cluster containing all genes for the conversion of caprolactam to adipate was detected by genome sequencing.

## Materials and methods

### Growth conditions

*Escherichia* coli C41 (DE3) cells (Lucigen, Halle-Zoersel, Belgium) were grown at 37 °C in an LB medium (Sambrook et al. 2001). When required, ampicillin (50 μg/mL) was added. The caprolactam-degrader *P. jessenii* GO3 was cultured at 30 °C in nitrogen-free minimal medium (MM) (Gabor et al. 2004), supplemented with caprolactam (4 mM) as carbon and nitrogen source. For the preparation of agar plates, the medium was supplemented with 2% agar or 1.6% H_2_O-rinsed agarose.

To determine the caprolactam tolerance of *P. jessenii* GO3, cells were precultured in MM supplemented with 0.1% caprolactam at 30 °C. Cells from these precultures were diluted 50- to 100-fold in 200 μL fresh medium supplemented with varying concentrations of caprolactam (0.05 to 1.2%). Subsequently, growth was monitored by measuring the absorbance at OD_600_ using a microplate spectrophotometer (PowerWave, BioTek, Winooski, VT, USA).

### Enrichment of caprolactam-degrading bacteria

For the isolation of bacterial strains, 1 g of residential grassland soil from Groningen (The Netherlands) was used to inoculate 50 mL nitrogen-free MM supplemented with 0.2% glucose and 0.5 mM caprolactam as sole carbon and nitrogen source. Cultures were incubated for 7 days in an orbital shaker at 30 °C. Subsequently, cells were transferred two times to fresh liquid medium after which pure cultures were isolated on MM agarose plates supplemented with 0.2% glucose and 0.5 mM caprolactam. To confirm growth of the resulting pure cultures on caprolactam, cells were reinoculated in selective liquid MM. Identification of isolated organisms was based on the analyses of the 16S rRNA gene sequence, received from the partial genome sequence obtained in this study, using the online EzTaxon database (Chun et al. 2007). *P. jessenii* strain GO3 is deposited at DSMZ (Braunschweig, Germany) under accession number DSM 106008.

### Genome sequencing and annotation

In order to obtain a partial genome sequence, total DNA from *P. jessenii* GO3 cells was isolated essentially as described previously (Poelarends et al. 1998; Sambrook et al. 2001). The resulting genomic DNA was subjected to paired end sequencing by BaseClear BV (Leiden, The Netherlands). The genome sequencing was done using a HiSeq2500 system (Illumina Inc., Eindhoven, The Netherlands). Sequencing of the GO3 genome yielded 1.7 million reads of ~ 100 bp in length. These were assembled by BaseClear, using the CLC Genomics Workbench version 7.0.4 (Qiagen, Venlo, The Netherlands), resulting in 1274 contigs with a total length of 7 mega base pairs (Mb). This whole genome project has been deposited at DDBJ/ENA/GenBank under the accession PDLL00000000. The version described in this paper is PDLL01000000. Subsequently, all generated contigs were used for automated annotation using the RAST server (Aziz et al. 2008).

### Molecular techniques

Standard recombinant DNA techniques were performed essentially as described previously (Sambrook et al. 2001). Restriction enzymes and polymerase were used according to the instructions of the supplier (New England Biolabs, Ipswich, MA, USA). Primers used in this study are listed in Table 1.

**Table 1:** Primers used

ATfw	TTCCTTCTCTAGAATGAACCAGTCAGTATCCTCGC
ATrev	TTCCTGAATTCTTAATGATGATGATGATGATGGCCGCCCGGACCAACCCACTGAGTGGTGTC
OPfw	ATCAAGCTTAATGAACACAGTAGACCCGATC
OPrev	AAGGAAAAAAGCGGCCGCTCAATGACCGGGAGTCAGTTC

### Plasmid constructions

For the purification of a 6-ACA aminotransferase (*Pj*AT), plasmid pET-PjAT was constructed containing an in-frame fusion of the *Pj*AT-encoding gene to a hexahistidine tag, behind the T7 promoter region. For this purpose, the *Pj*AT gene was amplified using *P. jessenii* GO3 chromosomal DNA and primers ATfw and ATrev. The resulting 1419 base pair product was then digested with *Nde*I/*Eco*RI and ligated into *Nde*I/*Eco*RI-digested pET20b^+^ (Novagen-Merck, Amsterdam, The Netherlands).

For the expression of caprolactamase subunits α and β, plasmid pET-OP was constructed containing both genes, behind the T7 promoter region. Since both genes are likely located in one operon, they were amplified together using *P. jessenii* GO3 chromosomal DNA and primers OPfw and OPrev. The resulting 3871 base pair fragment was then digested using *Hin*dIII and *Not*I and ligated into *Hin*dIII/*Not*I-digested pET20b^+^.

### Proteomics by mass spectrometry

*Pseudomonas jessenii* GO3 cells were cultured in 50 mL MM supplemented with 0.2% glucose and 5 mM (NH_4_)_2_SO_4_ or with 4 mM caprolactam. When the culture reached the late exponential growth phase, cells were harvested by centrifugation at 3000×*g* for 15 min. Cell pellets were washed once with 50 mM potassium phosphate buffer, pH 7.8, and stored at − 20 °C prior to use.

For mass spectrometry, cell pellets were resuspended in 50 mM potassium phosphate buffer, pH 7.8, and lysed using a Vibra Cell sonicator (Sonics, Newtown, CT, USA) at 0 °C. To remove unbroken cells and cell debris, the samples were centrifuged at 17,000×*g* for 60 min at 4 °C. Soluble protein was precipitated by the addition of 20% trichloroacetic acid (TCA). After incubating the samples for 1 h on ice, samples were centrifuged at 17,000×*g* for 30 min at 4 °C. Protein pellets were washed with ice-cold acetone to remove residual TCA. Dry protein extracts were then resuspended in 50 μL 50 mM NaOH. Reduction of the samples was performed with 5 μL of 500 mM dithiothreitol (DTT) in 350 μL 100 mM NH_4_HCO_3_ for 30 min at 25 °C, followed by derivatization of sulfhydryls by 30 min incubation at room temperature with 10 μL of 550 mM iodoacetamide. Trypsin digestion was performed overnight at 37 °C by addition of 4 μg trypsin gold (mass spectrometry grade, Promega (Leiden, The Netherlands)), followed by a second trypsin digestion for 3 h at 37 °C using 1 μg trypsin gold. Samples were prepared for injection by addition of 2.5% formic acid.

For LC-MS, peptides were first trapped on a precolumn (EASY-Column C18, 100 μm × 20 mm, 5 μm particle size, Thermo Scientific, Ermelo, The Netherlands) and separated on a capillary column (C18 PepMap 300, 75 μm × 100 mm, 3-μm particle size, Thermo Scientific) mounted on a Proxeon Easy-nLCII system (Thermo Scientific). Solutions of 0.1% formic acid in water, and 0.1% formic acid in 100% acetonitrile, were used as the mobile phases. A gradient from 5 to 40% acetonitrile was performed at a flow rate of 300 nL/min. Eluted peptides were analyzed using a linear ion trap Orbitrap hybrid mass spectrometer (LTQ-Orbitrap XL, Thermo Scientific). MS scans were acquired in the range from 300 to 2000 *m*/*z*. The five most intense ions per scan were selected for MS/MS fragmentation (35% normalized collision energy) and detected in the linear ion trap.

Peak lists were obtained from raw data files using Proteome Discoverer (version 1.1, Thermo Fisher Scientific). Mascot (version 2.1, Matrix Science, London, UK) was used for searching against the annotated *P. jessenii* GO3 genomic DNA sequence. Peptide tolerance was set to 10 ppm and the fragment ion tolerance to 2.0 Da, using semitrypsin as protease specificity and allowing for up to two missed cleavages. Oxidation of methionine residues, deamidation of asparagine and glutamine, and *S*-carboamidomethylation of cysteines were specified as variable modifications. The MS/MS-based peptide and protein identifications were further validated with the program Scaffold (version 4.6.1, Proteome Software Inc., Portland, OR, USA). Peptide identifications were accepted when the probability was greater than 95%. Protein identifications were based on at least two unique peptides identified by MS/MS, each with a confidence of identification probability higher than 99%.

For each growth condition, at least two replicates of two independent cultures were analyzed. Normalized intensity-based absolute quantification (iBAQ) values from Scaffold were used as a measure for the abundance of the identified proteins. Average iBAQ values were calculated for the different samples and subsequently log2 transformed. In case the protein was not detected, the log2-transformed iBAQ value was manually set to 8.5, 2.5-fold below the approximate limit of detection. The effect of growth conditions on specific protein amounts was calculated by dividing the average log2 iBAQ value for each protein in extracts from caprolactam-grown cells by the corresponding iBAQ value in protein extracts from control cells. A protein was considered upregulated when the log2-fold ratio was more than two and downregulated when the log2-fold ratio was less than 0.5.

The mass spectrometry proteomics data have been deposited to the ProteomeXchange Consortium via the PRIDE (Vizcaíno et al. 2016) partner repository with the dataset identifier PXD008544 and 10.6019/PXD008544.

### Expression and purification

The aminotransferase *Pj*AT and caprolactamase were both produced in *E. coli* C41 cells under control of the T7 promoter. *Pj*AT was expressed and purified as previously described for related aminotransferases (Palacio et al. 2016).

For the expression of the α and β subunits of caprolactamase, 0.5 mL of an overnight grown LB culture of pET-OP transformed cells was transferred to 50 mL autoinduction medium (ForMedium) containing ampicillin and incubated for 48 h in a rotary shaker at 17 °C. To prepare cell-free extracts, cells were washed in buffer A (50 mM ammonium bicarbonate, pH 8.5, 10 mM MgCl_2_), resuspended in buffer A, and lysed using a Sonics Vibra Cell sonicator at 0 °C. To remove unbroken cells and cell debris, the samples were centrifuged at 17,000×*g* for 30 min.

### Enzyme kinetics

To analyze aminotransferase activity in cell-free extracts, reactions were followed using HPLC analyses. Cell-free extracts were prepared in 50 mM potassium phosphate buffer (pH 8) containing 0.3 mM pyridoxal 5'-phosphate (PLP). Standard reaction mixtures contained 50 mM potassium phosphate buffer (pH 8), 2 mM amine donor (pyruvate or α-ketoglutarate), 5 mM 6-ACA, 0.3 mM PLP, and cell-free extract, in a total volume of 300 μL. Reactions were carried out at 28 °C. With different time intervals, 50 μL samples were taken and quenched by the addition of 50 μL 2 M HCl. After incubating the samples for 10 min on ice, samples were centrifuged for 10 min at 17,000×*g* and neutralized using 100 μL 1 M NaOH. Amino acids (6-ACA, alanine, glutamate) in the reaction mixtures were quantified by precolumn *o*-phthalaldehyde (OPA) derivatization and subsequent HPLC analyses. To this purpose, 10-μL sample was mixed with 40 μL 0.4 M boric acid (pH 9.7) and 10 μL OPA solution (Fisher Scientific) and incubated for 20 min at 30 °C. Then, 3 μL of the reaction sample were injected by an autosampler and analyzed by HPLC using a C18 OPA Adsorbosphere column connected to a Jasco FP-920 detector (excitation 350 nm; emission 450 nm). Compounds were eluted using a linear gradient (eluent A, 20 mM sodium acetate, pH 7.2, 0.5% (vol/vol) tetrahydrofuran, 0.018% (vol/vol) TEA and eluent B, 90% acetonitrile) at a flow rate of 0.5 mL/min.

The activity of purified *Pj*AT with pyruvate as the amine acceptor was estimated by coupling the reaction to alanine dehydrogenase and measuring the increase in NADH absorbance that occurs as a result of oxidative deamination of the produced alanine. Since pyruvate is a competitive inhibitor of alanine dehydrogenase, low pyruvate concentrations were used to minimize the lag time of the reaction. The concentration of alanine dehydrogenase in the assays was in excess (5 units/mL), to give aminotransferase*-*dependent velocities. Standard reaction mixtures contained 100 mM potassium phosphate buffer (pH 8), 5 U/mL alanine dehydrogenase, 2 mM NAD^+^, 0.05 mM PLP, 2 mM substrate, 0.2 mM pyruvate, and varying concentrations of enzyme in a total volume of 300 μL in flat-bottom 96-well microtiter plates. Reactions were carried out at 30 °C and analyzed using a microtiter plate reader (Synergy Mx Microplate Reader, BioTek Instruments, Bad Friedrichshall, Germany). Reaction mixtures lacking pyruvate (150 μL) were prewarmed before the reaction was initiated by the addition of 150-μL pyruvate solution. Each reaction was analyzed in triplicate. Initial rates were used to determine specific activities in units per mg protein (μmol/min/mg). Protein content was determined using the Bradford method, with bovine serum albumin as the standard.

The amination of 6-oxohexanoate with alanine as donor was also followed by coupling to alanine dehydrogenase. The reaction mixtures contained 100 mM potassium phosphate buffer (pH 8), 2 mM substrate, 0.1 mM NADH, 0.05 mM PLP, 8 U/mL alanine dehydrogenase, 5 mM ammonium bicarbonate, 5 mM l-alanine, and varying concentrations of enzyme in a total volume of 300 μL. Reactions were initiated by addition of 150 μL of l-Ala and carried out as described previously. Conversion was followed by measuring NADH depletion at 340 nm.

Caprolactamase activity was determined by analyzing reaction mixtures using an Acquity TQD mass spectrometer (Waters, Etten-Leur, The Netherlands). Standard reaction mixtures contained 2 mM substrate (caprolactam or 5-oxoproline), 5 mM ATP, 10 mM MgCl_2_, and cell-free extract, in 50 mM ammonium bicarbonate, pH 8.5. Samples were taken and quenched by the addition of 2% formic acid. Separation of the reaction content was performed by UPLC using a Waters Acquity UPLC HSS T3 Column (1.8 μm, 2.1 × 150 mm) and a linear gradient (eluent A: water, 0.1% formic acid; eluent B: 100 acetonitrile, 0.1% formic acid). The samples were analyzed in positive ion mode. To determine substrate reduction and product formation, multiple reaction monitoring (MRM) was performed, measuring the following fragments: caprolactam *m*/*z* = 96; 6-ACA *m*/*z* = 114; 5-oxoproline *m*/*z* = 84; glutamate *m*/*z* = 102.

## Results

### Isolation of bacterial strains using caprolactam as a sole nitrogen source

In order to isolate a bacterial strain possessing a caprolactam metabolism pathway, soil microorganisms were enriched for the ability to grow on caprolactam as sole nitrogen source. A pure culture was obtained by repeated transfer to fresh medium plates. The bacterial strain that was growing best on selective medium was used to study caprolactam metabolism in detail and was designated strain GO3.

**Fig. 1:**
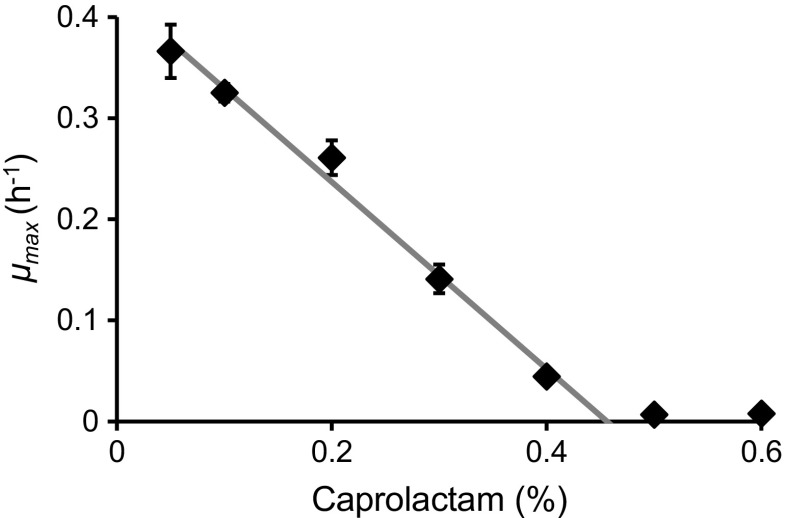
Specific growth rates of *P. jessenii* GO3 in media supplemented with different concentrations of caprolactam

Growth analyses revealed that strain GO3 was able to use caprolactam both as a sole nitrogen and carbon source. Interestingly, 6-aminohexanoic acid (6-ACA), a described intermediate in caprolactam degradation (Caspi et al. 2014), was not a possible growth substrate for this strain. In mineral medium (MM) supplemented with 0.05% caprolactam as sole carbon and nitrogen source, the calculated *μ*_max_ was 0.37 h^−1^. Growth analyses using different concentrations of caprolactam in MM revealed that the *μ*_max_ is reduced by higher concentration of caprolactam, with a calculated critical caprolactam concentration of 0.46% (Fig. 1).

### Draft genome sequence

After paired end sequencing of the genomic DNA from strain GO3, genome assembly resulted in 1274 contigs (N50: 10,682 bp), covering 6,993,317 bp. The GC content is 60% with 6231 predicted coding sequences. Among these predicted genes, 4754 were assigned a predicted function (76%). Furthermore, 3 rRNA and 61 tRNA genes were identified in the draft genome.

Analyses of the 16S rRNA gene revealed that the organism is a *Pseudomonas* species closely related to *P. jessenii* CIP 105274 (99.5% identity). The draft genome was compared to related *Pseudomonas* species of which the complete sequence is published, including the caprolactam-degrading organism *Pseudomonas mosselii* SJ10 (Park et al. 2014) (Table 2). Previous studies showed that in other caprolactam-utilizing *Pseudomonas* strains, the genes involved in caprolactam metabolism are plasmid localized (Boronin et al. 1984). Using gel electrophoresis of DNA extracts, we did not find a plasmid in *P. jessenii* GO3 (data not shown), suggesting a chromosomal location of the catabolic genes.

**Table 2:** General genomic features of various *Pseudomonas* species

General features	Pj GO3	Pb NFM421	Pk 1855–344	Pm SJ10
Size (Mb)	7.0	6.8	6.8	6.2
GC (%)	60.0	60.8	60.7	63.4
CDS	6231	6097	5856	5413
Protein with predicted function (%)	76.3			

The genome data are adopted from the original papers. These numbers may differ from numbers obtained with updated annotations

CDS coding sequences, Pb *Pseudomonas brassicacearum* NFM421 (Ortet et al. 2011), Pk *Pseudomonas kilonensis* 1855-344 (Eng et al. 2015), Pm *P. mosselii* SJ10 (Park et al. 2014)

### Identification of caprolactam degradation enzymes

A hypothetical caprolactam degradation pathway (Esikova et al. 2012) involves two unidentified enzymes: the ring-cleavage enzyme, presumably a hydrolase, and the enzyme involved in the deamination reaction, which could be an aminotransferase, an oxidase, or an amine dehydrogenase, all three producing an ω-ketoacid. To identify the enzymes involved in the pathway, the *P. jessenii* GO3 proteome was examined for caprolactam-induced proteins. To this purpose, *P. jessenii* cells were grown in minimal medium supplemented with caprolactam (4 mM), or glucose plus ammonium sulfate. Cell-free extracts were prepared from both cultures and subjected to quantitative proteome analysis using a label-free approach.

**Fig. 2:**
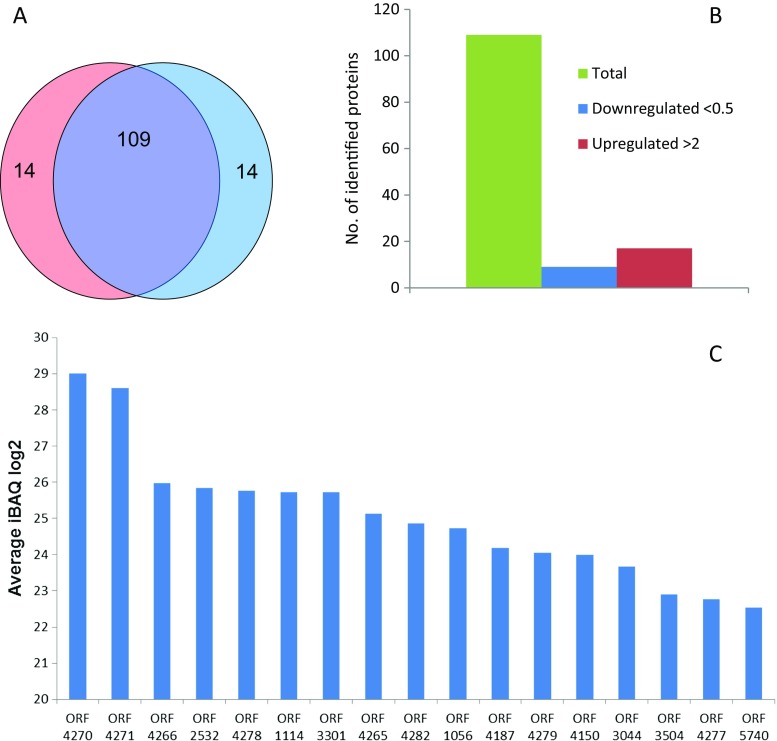
Proteomic analysis of *P. jessenii* GO3 cells, obtained from two independent replicate cultures. **A** Number of *P. jessenii* proteins identified by mass spectrometry in replicate culture 1 (red) and replicate culture 2 (blue). The overlapping region represents the number of proteins identified in both experiments (purple). **B** Bar graph representing the total number of identified *P. jessenii* proteins (green), including several caprolactam-induced proteins (red) and several caprolactam-repressed proteins (blue). A protein was considered upregulated when the protein level in caprolactam-induced cells divided by the protein level in non-induced cells was larger than two; a protein was considered downregulated when this ratio was smaller than 0.5. **C** Bar graph representing the average iBAQ log2 protein amounts of the identified upregulated proteins in caprolactam-grown cells. For all these hits, the protein amounts in non-induced cells were below the level of detection (not depicted in this plot) (Color figure online)

A total of 137 different proteins were identified in the combined independent replicate experiments, corresponding to 2.2% of the predicted *P. jessenii* proteome. Among these, 109 proteins were identified in both experiments and were subjected to further bioinformatic analysis (Fig. 2a). Seventeen of these proteins showed at least a 2-fold increase in log2 protein abundance in the caprolactam-grown cells as compared to the glucose cultures (Fig. 2b, Table 2; Supporting information Table S1 and Table S2). Interestingly, some proteins are highly induced on caprolactam, while others are just above the level of detection (LOD ~ 21, Fig. 2c). Conversely, in glucose-grown cells, these proteins were below the level of detection (iBAQ < 21, data not shown).

### Hypothetical caprolactam degradation pathway

**Fig. 3:**
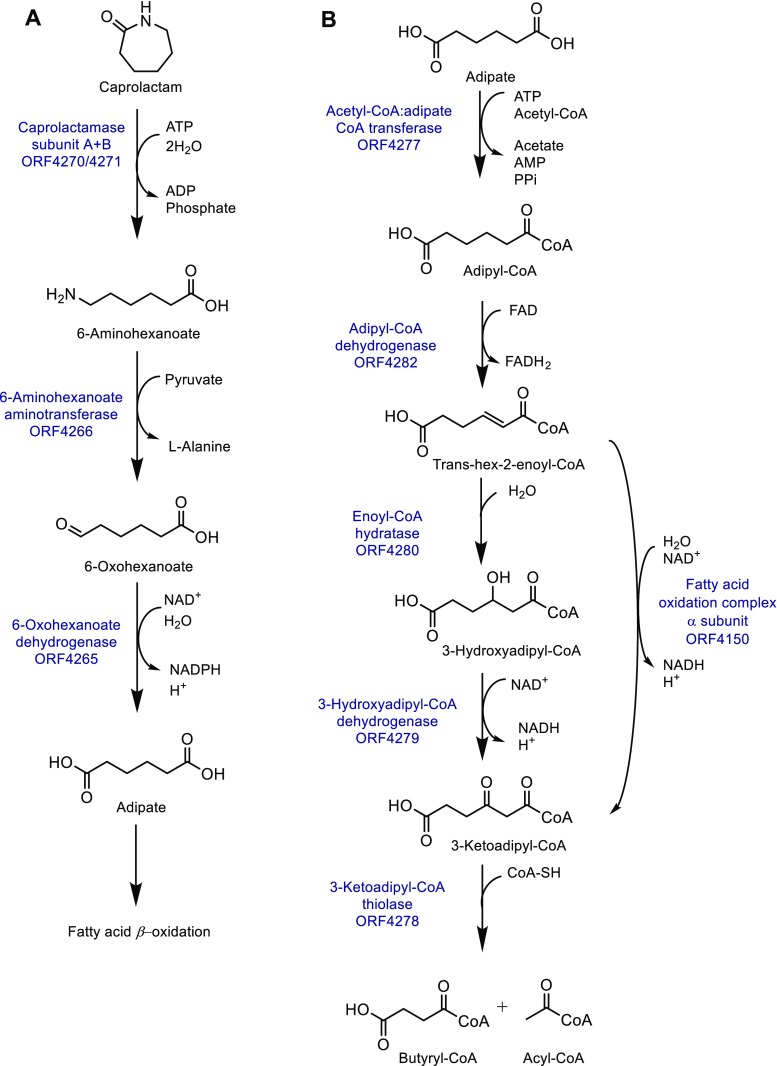
Hypothetical pathway for the biodegradation of caprolactam in *P. jessenii* cells. **A** The conversion of caprolactam to adipate. **B** The first steps of the *β*-fatty acid degradation

Based on the identified caprolactam-induced proteins (Table 3), in combination with previous data (Esikova et al. 2012), a complete putative *P. jessenii* caprolactam degradation pathway was built, including all enzymes that play a role in the pathway (Fig. 3). To enable growth on caprolactam as sole carbon and nitrogen source, an active uptake of caprolactam might be needed, which may be dependent on ABC transporter proteins. Four ABC transporter substrate binding proteins are significantly induced during growth on caprolactam, including ORF1056, ORF3044, ORF1114, and ORF2532 (Table 3). Database searches demonstrated homology to various ABC transporters, including the spermidine/putrescine binding proteins (ORF1056 and ORF3044), the branched chain amino acid binding proteins (ORF1114), and the amino acid binding proteins (ORF2532). Interestingly, ORF1114 and ORF2532 were predominantly induced when the cells were grown on caprolactam as sole carbon and nitrogen source (Fig. 2c). Possibly, one or several of these protein(s) are involved in the uptake of caprolactam.

**Table 3:** Caprolactam-induced proteins

Name	Contig	Size (bp)	Enzyme	Seq. identity to known proteins (%, organism)	EC number	Accession number
4265	5	1451	Succinate-semialdehyde dehydrogenase	81, *E. coli*	1.2.1.24	3JZ4
4266	5	1367	Omega aminotransferase	43, *V. fluvialis*	2.6.1.18	3NUI
4270	5	2114	Hydantoin utilization protein A	32, *Pseudomonas* sp.	3.5.2.-	Q01262
4271	5	1745	Hydantoin utilization protein B	30, *Pseudomonas* sp.	3.5.2.-	Q01262
4277	5	1238	Acetyl-CoA:oxalate CoA-transferase	39, *E. coli*	2.8.3.19	4HL6
4278	5	1202	Acetyl-CoA acetyltransferase	41, *Ralstonia eutropha*	2.3.1.9	4O99
4279	5	1541	3-Hydroxyacyl-CoA dehydrogenase	39, *R. eutropha*	1.1.1.35	4PZC
4282	5	1154	Acyl-CoA dehydrogenase	42, *Thermus thermophilus*	1.3.99.2	2DVL
2532	28	1031	ABC transporter, amino acid binding protein	59, *Brucella ovis*		4Z9N
3504	39	1922	Serine protein kinase	77, *E. coli*	2.7.11.1	P0ACY5
4150	48	2147	Fatty acid oxidation complex α subunit	94, *P. fragi*	4.2.1.17 5.3.3.8 1.1.1.35 5.1.2.3	1WDK
1056	157	1112	ABC transporter, putrescine binding protein	75, *P. aeruginosa*		3TTM
1114	161	1133	ABC transporter, branched chain amino acid binding protein	51, *Agrobacterium fabrum*		3IP5
3044	330	2549	ABC transporter, Spermidine/putrescine binding protein	31, *Streptococcus pneumoniae*		4EQB
3301	363	344	Putative enzyme of the cupin superfamily	98, *Pseudomonas* sp.		WP_084317822
4187	485	1325	Isocitrate lyase	82, *E. coli*	4.1.3.1	1IGW
5740	814	905	Histone H1-like protein HC2	43, *Chlamydia pneumoniae*		Q9Z8F9

Inside the cell caprolactam is most likely converted to 6-ACA. This conversion might be dependent on a caprolactamase, catalyzing the opening of the lactam ring. Inspection of the caprolactam-induced proteins for a lactamase-related protein resulted in the identification of two distinct polypeptides, ORF4270 and ORF4271. Database searches revealed identity to subunits A and B of the hydantoinase from *Pseudomonas* sp. (Table 3, respectively, 32 and 30% identity) and to the putative 5-oxoprolinase subunits A and B from *Pseudomonas putida* (OplA, 78% identity and OplB, 86% identity). The ORF4270 and ORF4271 encoded sequences also displayed weak similarity to eukaryotic 5-oxoprolinases which are known to catalyze the ATP-dependent hydrolytic decyclization of 5-oxoproline, producing l-glutamate (*Saccharomyces cerevisiae* OXP1/YKL215c, 22 and 23% identity, respectively) (Seddon et al. 1984). Since 5-oxoproline, hydantoin, and caprolactam share a lactam moiety, it is likely that proteins encoded by these two ORFs are involved in the caprolactamase reaction.

Further conversion of 6-ACA can proceed through deamination catalyzed by an aminotransferase, producing 6-oxohexanoate. Inspection of the caprolactam-induced proteins resulted in the identification of a protein with homology to the *Vibrio fluvialis* ω-amino acid aminotransferase (ORF4266, 43% identity). The well-characterized *V. fluvialis* enzyme catalyzes the pyruvate-dependent transamination of ω-amino acids and other amines to aldehydes or ketones (Shin et al. 2003).

The product 6-oxohexanoate would then be converted to adipate. In *Acinetobacter*, this reaction is catalyzed by a 6-oxohexanoate dehydrogenase (Iwaki et al. 1999). Among the caprolactam-induced proteins, one protein (ORF 4265) was identified with homology to the *E. coli* succinate-semialdehyde dehydrogenase. In *E. coli*, this enzyme converts succinate semialdehyde to succinate, which is part of the biodegradation of 4-aminobutyric acid (Donnelly and Cooper 1981). Since succinate-semialdehyde and 6-oxohexanoate are structurally similar, it is plausible that this enzyme is involved in the conversion of 6-oxohexanoate. Additionally, this protein has 38% sequence identity to the *Acinetobacter* 6-oxohexanoate dehydrogenase.

Finally, adipate most likely enters the *β*-oxidation pathway for degradation of fatty acids, which consists of multiple reactions (Janßen and Steinbüchel 2014). First adipate needs to be activated by CoA, resulting in adipyl-CoA. This reaction might be achieved by the caprolactam-induced protein with homology to the *E. coli* acetyl-CoA:oxalate CoA-transferase (ORF4277). In *E. coli*, this enzyme catalyzes the reversible conversion of oxalate and acetyl-CoA to oxalyl-CoA and acetate. Then, adipyl-CoA can be converted in a multistep process by means of an adipyl-CoA dehydrogenase (ORF4282), an enoyl-CoA hydratase, an 3-hydroxyadipyl-CoA dehydrogenase (ORF4279), and a 3-ketoadipyl-CoA thiolase (ORF4278). Homologs of all of these proteins were clearly induced in caprolactam-grown cells, where the enoyl-CoA hydratase function might be performed by a protein with homology to the *Pseudomonas fragi* fatty acid oxidation complex α subunit (ORF4150). In *Pseudomonas*, this complex catalyzes multiple reactions of the beta fatty acid oxidation, including the enoyl-CoA hydratase and the 3-hydroxyacyl-CoA dehydrogenase (Ishikawa et al. 2004).

### Gene organization

The sequence information for most caprolactam-induced proteins was found on contig 5, a large segment of 40,376 bp containing 36 putative open reading frames (ORF4261 to ORF4296). The genetic context of this contig was analyzed using the RAST server. This revealed that contig 5 comprises two gene clusters involved in the caprolactam degradation.

**Fig. 4:**
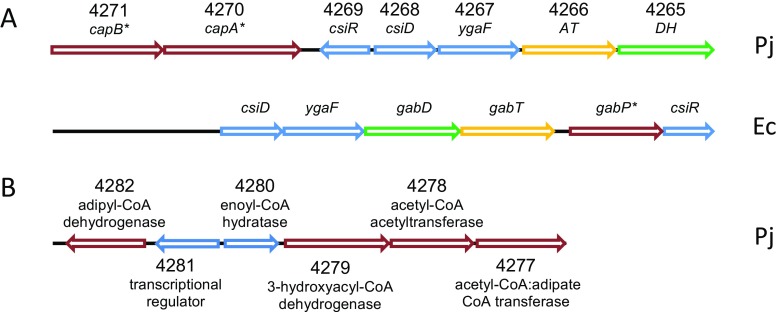
Schematic representation of the gene organization of contig 5, containing most of the caprolactam-induced genes, analyzed using the RAST server. **a** Gene cluster comprising the genes putatively involved in the conversion of caprolactam to adipate in *P. jessenii* (Pj) and a similar gene cluster with the genes involved in the conversion of *γ*-aminobutyrate in *E. coli* (Ec). Genes marked with an asterisk indicate clear differences between the *P. jessenii* and *E. coli* gene clusters. **b** Gene cluster comprising the genes putatively involved in the fatty acid β-degradation of adipate. Genes represented in blue: *P. jessenii* genes encoding proteins that were not significantly upregulated (Color figure online)

The first gene cluster contains the genes putatively involved in the conversion of caprolactam to adipate, including both subunits of the proposed caprolactam-induced caprolactamase (ORF4270, ORF4271), the omega aminotransferase (ORF4266), and a 6-oxohexanoate dehydrogenase (ORF4265) (Fig. 4a). In between these genes, three other ORFs are located (Fig. 4a, Pj, in blue). BLAST searches revealed that these open reading frames encode proteins with high homology to the l-2-hydroxyglutarate oxidase LhgO of *E. coli* (ORF4267, 72% identity), the starvation induced protein CsiD of *P. putida* (ORF4268, 75%), and the transcriptional regulator CsiR from *E. coli* (ORF4269, 59%). In *E. coli*, these genes cluster together with *gabD*, *gabT*, and *gabP* involved in the conversion of *γ*-aminobutyrate to succinate. The *gabD* gene encodes a succinate-semialdehyde dehydrogenase (DH), *gabT* a *γ*-aminobutyrate aminotransferase (AT) and *gabP* a *γ*-aminobutyrate permease. GabT and GabD represent similar catalytic activities as the *P. jessenii* omega aminotransferase (ORF4266) and the 6-oxohexanoate dehydrogenase (ORF4265), respectively. Clear differences between both gene clusters include (1) the absence of a homolog of the *γ*-aminobutyrate permease *gabP* in *P. jessenii*, which might explain why *P. jessenii* is not able to grow on minimal medium supplemented with 6-ACA as sole nitrogen source; (2) the absence of a homolog of the caprolactamase genes ORF4270 and 4271 in *E. coli*, which appeared to be involved in the first step in caprolactam degradation in *P. jessenii* (Fig. 4a, genes labeled with *).

The second gene cluster includes genes involved in the fatty acid *β*-oxidation, including the caprolactam-induced acetyl-CoA:oxalate CoA-transferase (ORF4277), acetyl-CoA acetyltransferase (ORF4278), 3-hydroxyacyl-CoA dehydrogenase (ORF4279), and butyryl-CoA dehydrogenase (ORF4282). In between these genes, two more open reading frames are located which according to BLAST searches encode for proteins with high homology to an enoyl-CoA hydratase from *Mus musculus* (ORF4280, 47% identity) and an IclR family transcriptional regulator from *Pseudomonas testosteroni* (ORF4281, 36%). A similar gene cluster containing homologs of all six genes is present in the genome of other bacteria (e.g., *Pseudomonas aeruginosa* PAO1), suggesting a wider occurrence of adipate metabolism by the same pathway.

### ATP-dependent caprolactamase

To confirm the presence of an ATP-dependent caprolactamase activity in *P. jessenii*, a cell-free extract of caprolactam-induced cells was prepared. Subsequently, the extract was incubated in the presence of caprolactam, ATP, and MgCl_2_, and the production of 6-ACA was examined by UPLC-MS. This revealed that caprolactam is indeed enzymatically converted to 6-ACA. When a similar assay was performed using the cell-free extract of glucose-grown *P. jessenii* cells, no conversion of caprolactam to 6-ACA was detected. This confirmed that the caprolactamase activity present in *P. jessenii* cells is induced during growth on caprolactam. Additionally, to study the ATP dependence of the putative caprolactamase in *P. jessenii* cells, cell-free extracts of caprolactam-grown cells containing MgCl_2_ were prepared and tested for the formation of 6-ACA in the absence of ATP. No 6-ACA formation was detected, demonstrating that ATP indeed is required for the enzymatic hydrolyses of caprolactam to 6-ACA.

**Fig. 5:**
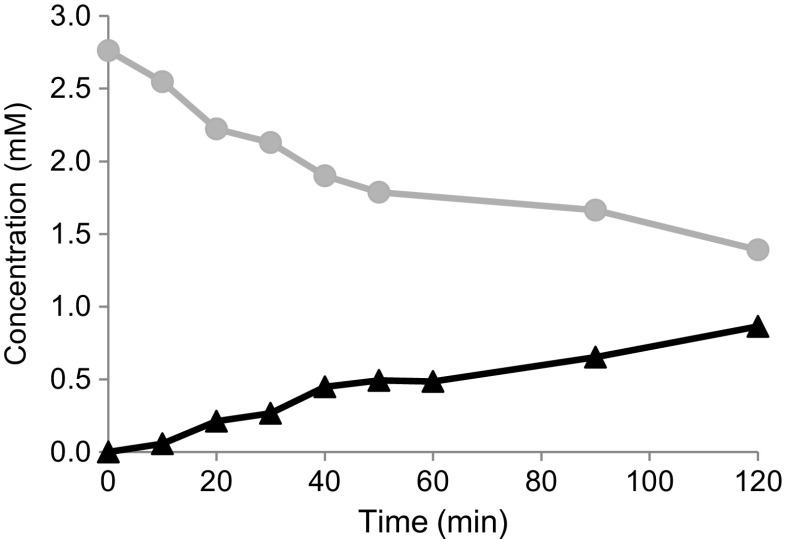
UPLC-MS analyses monitoring the formation of 6-ACA (black line, triangles) and the degradation of caprolactam (grey line, closed circles). The reaction mixtures contained 2 mM caprolactam, 5 mM ATP, 10 mM MgCl_2_, and 75 μg/mL cell-free extract, in 50 mM ammonium bicarbonate, pH 8.5

To confirm the role of the putative caprolactamase (ORF4270, ORF4271) in the conversion of caprolactam, the genes were expressed in *E. coli* C41. To express both subunits (CapA, CapB) simultaneously, the entire operon including the 36-bp intergenic region (Fig. 4) was cloned under control of a single T7 promoter. Expression in *E. coli* resulted in the high-level production of two proteins of the expected size, of which approximately 60% was present in the soluble fraction. UPLC-MS analysis was performed to detect the formation of 6-ACA in a mixture containing caprolactam, ATP, MgCl_2_, and *E. coli* cell-free extract. Time course analyses demonstrated that 6-ACA levels increased and caprolactam levels decreased in time. A specific activity of 0.14 U/mg was calculated for the *E. coli* cell-free extract (Fig. 5). In reaction mixtures containing caprolactam, ATP, MgCl_2_, and *E. coli* extract from cells not producing the caprolactamase, no detectable 6-ACA was observed even after 3 h of incubation. Thus, the caprolactamase activity detected in the induced *E. coli* (pET-OP) extract originates from the expressed α and β subunits of the caprolactamase. Since the sequence of the α and β subunits showed homology to enzymes annotated as 5-oxoprolinase, reaction mixtures containing 5-oxoproline, ATP, MgCl_2_, and *E. coli* cell-free extract were tested for the production of glutamate. UPLC-MS analyses demonstrated no detectable glutamate even after 3 h of incubation. This revealed that 5-oxoproline is not a substrate for the identified *P. jessenii* caprolactamase.

### Characterization of the omega aminotransferase

Most omega aminotransferases are PLP-fold type I enzymes that catalyze the transfer of an amino group from a β-, γ- or other ω-amino acid or an amine to pyruvate or α-ketoglutarate (Schiroli and Peracchi 2015). In order to confirm the presence of 6-ACA ω-aminotransferase activity in *P. jessenii* GO3, a protein extract of caprolactam-induced cells was prepared and incubated with 6-ACA and pyruvate or α-ketoglutarate, and levels of 6-ACA and produced alanine or glutamate were determined using OPA derivatization and HPLC. This revealed that caprolactam was indeed enzymatically converted with pyruvate as the amino acceptor. In the absence of pyruvate, no conversion of 6-ACA was detected. When a similar assay was performed using cell-free extract of glucose-grown *P. jessenii* cells, no conversion of 6-ACA or pyruvate was found. This confirmed the presence of a caprolactam-inducible ω-transaminase activity in *P. jessenii* GO3 cells.

To establish if this ORF4266-encoded putative ω-amino acid aminotransferase (*Pj*AT) is responsible for the conversion of 6-ACA, activity assays were performed using *E. coli*-expressed *Pj*AT. Since *E. coli* contains multiple aminotransferases, *Pj*AT was equipped with a C-terminal His6-tag, and the enzyme was purified using a Ni-NTA resin. This yielded ca. 30–35 mg purified enzyme/L of culture. The activity of the enzyme was examined in both directions, so with 6-oxohexanoate or with 6-ACA as substrate, together with l-alanine or pyruvate as amino acceptor. Activities were obtained by coupling the reaction to that of alanine dehydrogenase and following production or consumption of NADH spectrophotometry at 340 nm. This revealed a specific activity of 0.2 U/mg using 6-ACA and pyruvate as the substrates and an activity of 4.5 U/mg using 5-oxohexanoate and alanine as the substrates. These activities would suffice to enable strain GO3 to use caprolactam as a nitrogen source for growth.

## Discussion

In this work, we explored the caprolactam degradation pathway in the bacterium *P. jessenii* strain GO3. Previous studies suggested an overall catabolic pathway for caprolactam metabolism, but the enzymes catalyzing the first two steps, i.e., conversion of caprolactam to 6-oxohexanoate, remained unknown. Using proteomic studies, we identified an ATP-dependent lactamase involved in the conversion of caprolactam to 6-ACA and an ω-aminotransferase responsible for the subsequent conversion of 6-ACA to 6-oxohexanoate. Additionally, we identified various other enzymes and genes putatively involved in the caprolactam catabolic pathway.

The ATP-dependent lactamase catalyzing caprolactam ring opening was identified by proteomic studies, sequence comparison to known proteins with lactamase activity, and functional expression in *E. coli*. Sequence comparison indicated similarity to proteins annotated as 5-oxoprolinase or hydantoinase. Oxoprolinases catalyze the ATP-dependent hydrolysis of 5-oxoproline to glutamate (EC 3.5.2.9). The sequence of only four oxoprolinases with confirmed activity has been reported, including the enzymes from rat and cow (Ye et al. 1996; Watanabe et al. 2004), *S. cerevisiae* (Kumar and Bachhawat 2010), and the human enzyme. The latter can harbor mutations of medical relevance (Calpena et al. 2015). The eukaryotic 5-oxoprolinases are homodimers with subunits of approximately 137 kDa, whereas the *P. putida* 5-oxoprolinase, of which the sequence is not reported, consists of two different subunits of approximately 75 and 63 kDa (Seddon and Meister 1986; Li et al. 1988). Subunit A, which is homologous to the N-terminal part of the 137 kDa subunit of the eukaryotic enzymes, catalyzes the phosphorylation of enzyme-bound 5-oxoproline, whereas subunit B, which is homologous to the C-terminal part of eukaryotic oxoprolinase, is required for hydrolysis of the phosphorylated hydroxypyrrole. Other enzymes with significant sequence similarity to the caprolactamase are the ATP-dependent hydantoinases. Only the N-terminal sequences of the two subunits of the enzyme isolated from *P. putida* 77 have been reported (Ogawa et al. 1995), but BLAST searches allow retrieval of complete sequences from *Marinobacterium profundum* (WP_067296627.1 and WP_067296623.1). Sequence alignments show that in addition to the A and B subunits of the bacterial oxoprolinases mentioned previously, also the A and B subunits of these putative hydantoinases and the α and β subunits of caprolactamase described here correspond to the N-terminal part and C-terminal part, respectively, of the eukaryotic oxoprolinases. All these ATP-dependent hydrolases belong to InterPro families IPR002821 and IPR003692. We demonstrated the ATP dependence of the caprolactamase activity using enzyme expressed in *E. coli*. Within the families, there are clear differences. For example, the substrate range of the *P. jessenii* caprolactamase lacks 5-oxoprolinase activity. It seems possible that many bacterial genes annotated in GenBank as 5-oxoprolinase actually are (capro)lactamase genes or cyclic dipeptide hydrolase genes, since there is a higher sequence similarity to the CapA and CapB ORFs than to confirmed oxoprolinases.

Based on sequence and structural analysis, these lactamases can be further grouped with the ATP-dependent carboxylases/lactamase superfamily, which includes carboxylases acting on acetone and acetophenone (Weidenweber et al. 2017). The structure of the acetophenone carboxylase from *Aromatoleum aromaticum* EbN1 was recently solved (pdb 519w), revealing its quaternary structure as (αα′βγ)_2_. Sequence motifs indicative of ATP binding are conserved between the α subunit of the carboxylase and the α subunit of caprolactamase (ORF 4270), and the β subunit of the carboxylase is homologous to the β subunit of caprolactamase (ORF 4271). Thus, the elucidation of the caprolactam catabolism described here adds a new member to this diverse group of ATP-dependent hydrolytic enzymes. By similarity to oxoprolinase and hydantoinase, caprolactam hydrolysis by the caprolactamase is expected to proceed by phosphorylation of the enol (lactim) tautomer mediated by the α subunit, followed by its hydrolysis with a role for the β subunit. Structures of lactamases that would provide detailed insight are lacking, however.

The 6-aminohexanoate aminotransferase (*Pj*AT) catalyzing the second step in caprolactam catabolism was also identified by proteomic studies, sequence similarities, and functional overexpression in *E. coli*. BLAST searches using the protein sequence of the *Pj*AT demonstrated relatedness to the fold-type I PLP enzymes, more in particular to the subgroup II aminotransferases, which are now often grouped as class III aminotransferases (Schiroli and Peracchi 2015; Steffen-Munsberg et al. 2015). These enzymes catalyze conversion of ω-amino acids to aldehydes (EC 2.6.1.18). In vitro characterization of *Pj*AT confirmed the expected deamination of 6-ACA to 6-oxohexanoate. The enzyme was also active in the reverse reaction: l-alanine-dependent amination of 6-aminohexanoate. This conversion is of potential interest for a biosynthetic 6-ACA production pathway that was recently engineered into *E. coli* (Turk et al. 2015). Comparison with the *V. fluvialis* aminotransferase, which is the closest well-studied homolog, revealed conservation of several residues important for proper activity (PDB code 4E3Q; (Midelfort et al. 2013)). The lysine required for the formation of the internal aldimine (Schiff base) with the PLP cofactor is present at a conserved position. Further work on the biocatalytic and structural properties of the enzyme is ongoing. The *Pj*AT aminotransferase has 27.3% pairwise sequence identity with the NylD1 aminotransferase recently described in the nylon oligomer degrader *Arthrobacter* sp. KI72 (Takehara et al. 2018). The closest homolog of the latter enzyme in the *P. jessenii* genome is ORF2380 (48.6% pairwise identify), but an upregulation of this protein was not detected by proteomic analysis of caprolactam-grown *P. jessenii* cultures.

Previously, the full genome of *P. mosselii*, another caprolactam degrading organism isolated from wastewater of a nylon producing industrial complex in Korea, was sequenced by Park and coworkers (Park et al. 2014). Interestingly, BLAST searches using the sequences of the genes that were found here to be involved in caprolactam degradation against the full *P. mosselii* genome sequence revealed genes with high similarity to these proteins (aminotransferase 100%, CapA 99.6%, and CapB 99.3% sequence identity). This suggests the same caprolactam degradation pathways in both *Pseudomonas* species. Additionally, BLAST searches against the non-redundant protein database revealed highly similar open reading frames in annotated sequences of various other *Pseudomonas* strains, indicating that the ability to hydrolyze lactams or cyclic peptides may not be unusual in *Pseudomonas*. Other nylon by-products that can be degraded by microorganisms include 6-ACA dimers, cyclic dimers, and oligomers. Metabolism is dependent on hydrolases, such as NylA, NylB, and NylC, which have different specificities (Negoro 2000). NylA catalyzes the hydrolysis of 6-ACA cyclic dimer, resulting in the formation of the 6-ACA dimer, which can be converted by NylB, generating 6-ACA. NylC catalyzes the hydrolysis of 6-ACA oligomers. BLAST searches using *Flavobacterium* sp. NylA, B, and C revealed that *P. jessenii* contains homologs of NylA and NylB, but not of NylC, suggesting the absence of a complete pathway for metabolism of 6-ACA polymers.

## Electronic supplementary material

ESM 1 (PDF 173kb)
