# Supplementary material for: Characterization of the caprolactam degradation pathway in Pseudomonas jessenii using mass spectrometry-based proteomics
Source: Appl Microbiol Biotechnol. 2018 May 31;102(15):6699–711. doi: 10.1007/s00253-018-9073-7 (PMC6061476; doi:10.1007/s00253-018-9073-7)
Supplement: Supplementary file 1 — (PDF 173kb) [file 253_2018_9073_MOESM1_ESM.pdf]

Supplementary data

Applied Microbiology and Biotechnology

Characterization of the caprolactam degradation pathway in *Pseudomonas jessenii* using mass spectrometry-based proteomics

Marleen Otzen, Cyntia Palacio, Dick B. Janssen*
Department of Biochemistry, Groningen Biomolecular Sciences and Biotechnology Institute, University of Groningen, The Netherlands

*Corresponding author
Department of Biochemistry,
Groningen Biomolecular Sciences and Biotechnology Institute,
University of Groningen,
Nijenborgh 4,
9747 AG Groningen,
The Netherlands
E-mail: d.b.janssen@rug.nl
Fax: 0031 50 36 34165
Phone: 0031 50 35 34008.

Table S1: Protein abundances in cell free extracts of caprolactam-grown *P. jessenii* cells determined by LC-MS/MS.

ORF	Mass	Database source	Sequence coverage (average)	Total spectrum count (average)	iBAQ (average)	iBAQ_log2 (average)
ORF 4270	76 kDa	fig|6666666.92865.peg.4270	0.39	102	5.40E+08	29.01
ORF 4271	63 kDa	fig|6666666.92865.peg.4271	0.319	41.8	4.08E+08	28.61
ORF 4266	50 kDa	fig|6666666.92865.peg.4266	0.219	16	7.12E+07	25.15
ORF 2532	37 kDa	fig|6666666.92865.peg.2532	0.20	8.6	5.99E+07	25.83
ORF 4278	41 kDa	fig|6666666.92865.peg.4278	0.24	9	5.86E+07	25.75
ORF 1114	40 kDa	fig|6666666.92865.peg.1114	0.25	10.6	6.14E+07	25.73
ORF 3301	13 kDa	fig|6666666.92865.peg.3301	0.16	1.8	5.80E+07	25.72
ORF 4265	52 kDa	fig|6666666.92865.peg.4265	0.19	12.4	3.92E+07	25.13
ORF 4282	42 kDa	fig|6666666.92865.peg.4282	0.12	6.4	3.12E+07	24.85
ORF 1056	40 kDa	fig|6666666.92865.peg.1056	0.18	6.6	2.82E+07	24.73
ORF 4187	49 kDa	fig|6666666.92865.peg.4187	0.079	4	1.91E+07	24.18
ORF 4279	55 kDa	fig|6666666.92865.peg.4279	0.096	3.8	1.75E+07	24.06
ORF 4150	77 kDa	fig|6666666.92865.peg.4150	0.15	8.8	1.69E+07	23.99
ORF 3044	38 kDa	fig|6666666.92865.peg.3044	0.14	3.8	1.45E+07	23.66
ORF 3504	74 kDa	fig|6666666.92865.peg.3504	0.062	4	7.85E+06	22.90
ORF 4277	44 kDa	fig|6666666.92865.peg.4277	0.073	2.8	7.12E+06	22.76
ORF 5740	30 kDa	fig|6666666.92865.peg.5740	0.11	6.8	6.46E+06	22.53

Values represent average characteristics of all identified protein. This data is based on 2 independent replicate cultures, analyzed in duplo or triplo by LC-MS/MS. None of these hits was detectable in glucose-grown cells.

Table S2: Difference in iBAQ-log2 protein level in caprolactam-grown cells compared to glucose/ammonium sulfate-grown cells.

	iBAQ Cap/iBAQ gluc
ORF 4270	3.41
ORF 4271	3.37
ORF 4266	2.96
ORF 2532	3.04
ORF 4278	3.03
ORF 1114	3.03
ORF 3301	3.03
ORF 4265	2.96
ORF 4282	2.92
ORF 1056	2.91
ORF 4187	2.84
ORF 4279	2.83
ORF 4150	2.82
ORF 3044	2.78
ORF 3504	2.69
ORF 4277	2.68
ORF 5740	2.65
